# The 12-CK Score: Global Measurement of Tertiary Lymphoid Structures

**DOI:** 10.3389/fimmu.2021.694079

**Published:** 2021-06-29

**Authors:** Roger Li, Anders Berglund, Logan Zemp, Jasreman Dhillon, Ryan Putney, Youngchul Kim, Rohit K. Jain, G. Daniel Grass, José Conejo-Garcia, James J. Mulé

**Affiliations:** ^1^ Department of Genitourinary Oncology, H. Lee Moffitt Cancer Center, Tampa, FL, United States; ^2^ Department of Immunology, H. Lee Moffitt Cancer Center, Tampa, FL, United States; ^3^ Department of Biostatistics and Bioinformatics, H. Lee Moffitt Cancer Center, Tampa, FL, United States; ^4^ Department of Pathology, H. Lee Moffitt Cancer Center, Tampa, FL, United States; ^5^ Department of Radiation Oncology, H. Lee Moffitt Cancer Center, Tampa, FL, United States

**Keywords:** tertiary lymphoid structures, 12-CK score, immune checkpoint blockade, prognostic biomarker, predictive biomarker

## Abstract

There is emerging evidence that the adaptive anti-tumor activity may be orchestrated by secondary lymphoid organ-like aggregates residing in the tumor microenvironment. Known as tertiary lymphoid structures, these lymphoid aggregates serve as key outposts for lymphocyte recruitment, priming and activation. They have been linked to favorable outcomes in many tumor types, and more recently, have been shown to be effective predictors of response to immune checkpoint blockade. We have previously described a 12-chemokine (12-CK) transcriptional score which recapitulates an overwhelming enrichment for immune-related and inflammation-related genes in colorectal carcinoma. Subsequently, the 12-CK score was found to prognosticate favorable survival in multiple tumors types including melanoma, breast cancer, and bladder cancer. In the current study, we summarize the discovery and validation of the 12-CK score in various tumor types, its relationship to TLSs found within the tumor microenvironment, and explore its potential role as both a prognostic and predictive marker in the treatment of various cancers.

## Introduction

Traditionally, effective adaptive immune response against cancer requires the rendezvous between the tumor antigen/major histocompatibility complex expressed on mature dendritic cells (DC) traveling from the primary tumor site and the resident CD4^+^ and CD8^+^ T cells in the secondary lymphoid organs. Here, naïve CD8^+^ T cells are primed and upregulate homing receptors that bind to cognate ligands expressed on inflamed vasculature, enabling entry into peripheral tissue (1). B cells are concurrently activated in the secondary lymphoid organs upon antigen binding and receive help from T follicular helper cells (T_fh_) to proliferate and form a secondary follicle, which progressively becomes a germinal center that persists until antigenic clearance. Within the germinal center, B cells undergo several processes including somatic hypermutation (SHM), affinity maturation, and class switching to allow production of antibodies with increasing affinity for the cognate antigen (2). These B cells eventually give rise to plasma cells that secrete higher-affinity and class-switched antibodies in the latter part of the primary immune response, or into memory B cells to coordinate the secondary immune response upon re-insult (2).

There is emerging evidence that adaptive anti-tumor immunity can also be orchestrated at secondary lymphoid organ-like aggregates within the tumor microenvironment (TME) called tertiary lymphoid structures (TLS) (3, 4). TLSs were first described in chronic inflammatory conditions, such as infection, autoimmune disease, and organ transplant rejection (2). They are posited to be 1) the gateway of naïve lymphocyte infiltration into the TME; 2) privileged sites for coordinated tumor antigen presentation and lymphocyte priming, differentiation, and proliferation, leading to a robust tumor-specific immune response. In line with these hypotheses, preclinical work has demonstrated the ability of adoptively transferred naïve CD8^+^ T cells to directly enter the TME through interactions with TLS-associated high endothelial venules (HEVs) in mice devoid of secondary lymphoid organs (5). Subsequently, their differentiation into functional effectors led to improved cancer control (6). Spatially, an enriched population of naïve CD8^+^ T cells was found to reside within the TLS, while effector memory CD8^+^ T cells were predominantly found in the tumor stroma (6). Furthermore, activated CD38^+^ and CD69^+^ tumor infiltrating T lymphocytes were enriched in TLS^Hi^ tumors, implicating TLS to be the site of T cell priming and activation (7). On the other hand, there is also evidence that TLS is capable of supporting functional germinal centers to promote affinity maturation and differentiation of B cells, leading to enhanced humoral response (2). Expression of activation-induced cytidine deaminase, a marker for SHM, has been described in TLS B cells within the context of autoimmunity, infection, allograft rejection, and cancer (8). Moreover, restricted profile of variable (V)-gene repertoire usage, highly mutated V regions and oligoclonal diversification of infiltrating B cells and plasma cells found within TLS^Hi^ samples serve as circumstantial evidence for SHM taking place within the germinal centers of the TLS (8).

Consistent with their putative role in lymphocyte recruitment and activation, TLS has been found to be a favorable prognostic indicator in several tumor types (3, 7, 9–13). Although often appearing in tumors with high T cell infiltrates, the absence of TLS in tumors otherwise heavily infiltrated by T cells was associated with inferior prognosis compared to those with high TLS (7, 14). In addition, a trio of recently published studies linked the presence of TLS within the TME to increase efficacy from immune checkpoint blockade (ICB) therapy in melanoma, renal cell carcinoma and soft-tissue sarcoma (14–16). Since, the clinical benefits of TLS within the TME has also been recapitulated in the setting of immunotherapy in other tumor types (17).

## Transcriptomic Signatures

With its diverse clinical prognostic and predictive implications, several classification schemes have been proposed to semi-quantitatively assess the presence, complexity and density of the TLS within the TME (18–20). Several of these schemes have coalesced on three classes: early TLS composed of dense lymphocytic aggregates without follicular dendritic cells; primary follicle-like TLS having FDCs but no germinal center reaction; and secondary follicle-like TLS, having an active GC reaction (18, 21, 22). However, given the heterogeneity of their histology and spatial distribution, systematic evaluation of the TLS within the TME is difficult. Alternatively, we and others have leveraged bulk RNA expression data to identify signatures associated with TLS enrichment. These signatures are either related to chemokine expression and/or cell populations found within the TLS.

Based on their observation that the presence of follicular helper T (Tfh) cells within the TME were critically linked to robust tumoral immune infiltration and TLS formation, Gu-Trantien et al. (10) devised an 8-gene Tfh signature to reflect the presence of TLS. They subsequently found this signature to be prognostic in both breast cancer patients undergoing surgical resection with or without neoadjuvant chemotherapy. Amongst its components, CXCL13 expression was found to have the closest association with tumor immune infiltration and the driver of the prognostic value of the gene signature. In addition to its prognostic value in breast cancer, *CXCL13* has also been found to enable identification of TLS in colorectal cancer (23) and soft tissue sarcoma (16). In gastric cancer, Tbet+ T cells and CD20+ B-cell follicles were associated with improved relapse-free survival, serving as rationale for a coordinated Th1 and B cell stromal gene signature, which was found to predict for presence of TLS along with improved cancer specific survival (24). Finally, Cabrita et al. used differential expression analysis from melanoma samples with and without TLS to construct a gene signature consisting of B-cell specific genes such as *CD79B* and *CCR6*, TLS-hallmark genes like *CCR7*, *CXCR5* and *SELL*, as well as *CXCL13*. This TLS-signature was found to be prognostic amongst metastatic melanoma patients within TCGA and also predictive of prolonged survival following treatment with CTLA4 blockade (14). Although promising, these transcriptomic signatures have not been thoroughly examined across tumor types and may be influenced by unique expression profiles within the TME of their origin. In contrast, the most well studied transcriptomic signature recapitulating the presence of TLS is the 12-chemokine score.

### 12-CK Score - Origins

The 12-chemokine (12-CK) score emerged from an in-depth analysis of the immune gene expression data in colorectal carcinoma and its correlation with patterns and compositions of lymphoid infiltrates (25). Using Affymetrix microarray data derived from 326 colorectal carcinoma samples, Coppola et al. identified a metagene grouping with overwhelming enrichment for immune-related and inflammation-related genes. On histologic review, tumors highly expressive of this metagene grouping exhibited robust peri-tumoral inflammatory reactions accentuated by the presence of TLS (25). These structures contained both B and T lymphocytes, as well as CD21^+^ dendritic cells within their germinal centers, establishing their true follicular nature. Furthermore, heat mapping analysis revealed a strong correlation between the chemokine genes and TLS-enriched tumors. Hierarchical clustering of tumors with and without TLS was performed on a selected set of chemokine genes, which was found to also closely associate with the immune-related metagene grouping. For each gene, a single representative probe set with the highest dynamic range across all profiled samples was picked up from all probe sets that mapped to a given gene symbol. Genes were then clustered using Pearson’s correlation distance metric resulting in the final 12-CK score consisting of CCL2, CCL3, CCL4, CCL5, CCL8, CCL18, CCL19, CCL21, CXCL9, CXCL10, CXCL11, and CXCL13. This signature was found to be independent of tumor stage, location, microsatellite instability status and treatment received. More importantly, the 12-CK score significantly prognosticated for improved overall survival.

That the presence of TLS was associated with a high chemokine expression signature was not surprising. While the precise sequence of TLS development has yet to be elucidated, there is now evidence demonstrating clear involvement of certain chemokine signaling pathways. Lymphotoxin (LT)-α/β are essential for the establishment and maintenance of lymphoid structures (26). Specifically, signaling through the LTβ Receptor (LTβR) is required for HEV differentiation and the formation of organized lymphoid aggregates (27). LT-α/LT-β also induce the production of CCL19, CL21, and CXCL13 through positive feedback loops, in which chemokine-producing cells expressing LTβR recruit further B cell infiltration, leading to increased production of LT-α/LT-β and in turn, further LTβR stimulation (28, 29).

LIGHT is another lymphotoxin-related cytokine expressed by T cells, immature DC, and macrophages that plays a critical role in the recruitment of CD8^+^ T lymphocytes and their subsequent proliferation and differentiation (30). In addition, LIGHT synergizes with IFN-γ to enhance the production of CXCL9, CXCL10, and CXCL11, which serve to recruit and polarize CXCR3^+^ mediated T_H_1 response (31). LIGHT also actively recruits NK cells to the site of inflammation, which in turns produces various cytokines leading to T cell infiltration (32) and DC maturation (33). In the context of tumor immunology, LIGHT has been demonstrated to broadly convey antitumor effects in a diverse range of malignancies, including fibrosarcoma (34), melanoma (35), B cell lymphoma (36), cervical cancer (37), and breast cancer (35). Finally, LIGHT has recently been shown to trigger TLS assembly *in vivo* by inducing the production of CCL21 by tumor endothelial cells and to promote the influx of endogenous T cells. Combination therapy using LIGHT and checkpoint inhibition was able to overcome immune resistance observed in autochthonous pancreatic tumors (38).

CCL19 and CCL21 are important chemokines constitutively expressed by stromal cells that recruits CCR7^+^ cells to the site of inflammation (39). B cell chemotaxis mediated through a CCL21 gradient is enhanced in the presence of Type-I IFN-α, which acts to decrease the ligand induced receptor internalization of CCR7 (receptor for CCL21), thereby allowing more efficient B cell trafficking within the pro-inflammatory TME (40). Ectopic expression of CCL19 or CCL21 in pancreatic islets led to organized lymphocytic infiltrates containing HEVs and stromal cells, resembling TLS (41). Furthermore, in the context of melanoma, treatment using DCs engineered to express recombinant CCL21 led to the recruitment of naïve T cells to the site of vaccination as well as increased formation of TLS (42). Through interactions between the naïve T cells and the engineered DC at the site of vaccination, the primary immune response was initiated and escalated into a more powerful systemic antitumor immunity, culminating in the regression of local and metastatic lesions (42).

Another chemokine known to play a critical role in the formation of TLS is CXCL13, through its interactions with CXCR5. Mice deficient in CXCL13 or CXCR5 lack follicular DC network and are thus devoid of any structured lymphoid organs, including lymph nodes, Peyer’s patches, and spleen (28). CXCR5 is upregulated on DC and CD4^+^ T cells in response to an infectious stimulus, promulgating the infiltration of CXCL13 expressing B cells to immune priming sites and their subsequent activation and antigen presentation (43, 44). Luther et al. showed that overexpression of CXCR5 alone was sufficient to induce the formation of TLS consisting of B and T cell zones, HEVs, and stromal cells (45).

Together, current understanding of the chemokine mediated cellular trafficking strongly support using the 12-CK score as the definitive biomarker for TLS formation. High 12-CK scores within the TME also indicates robust immunogenic activity and may serve as a marker for powerful pre-existing immunosurveillance.

### Pan-Cancer 12-CK Expression Analysis

**Figure 1:**
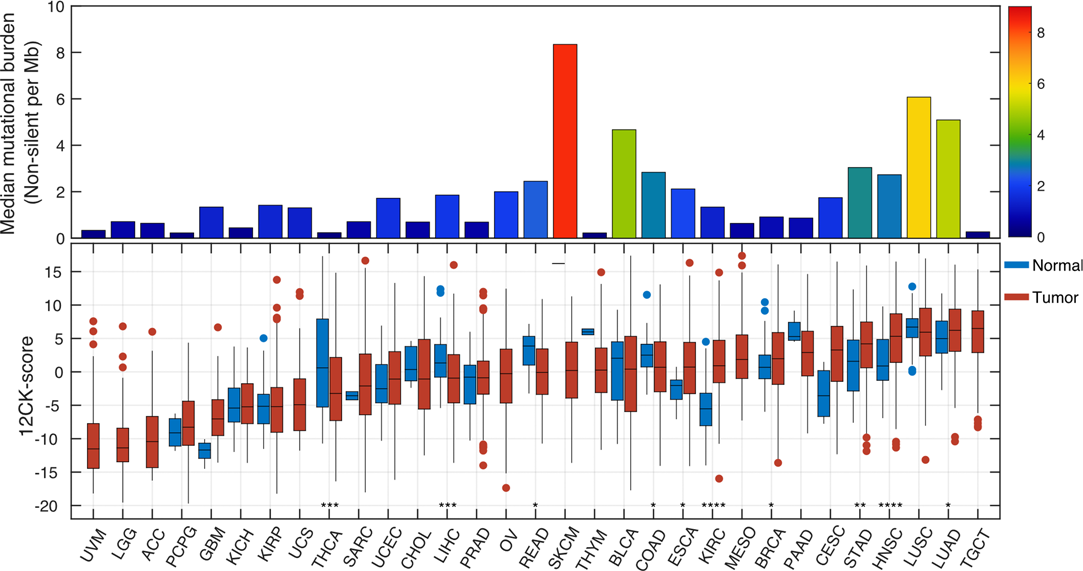
Pan-cancer analysis of the 12-chemokine score. The 12-CK score was extracted from the RNA expression data from various tumor types within The Cancer Genome Atlas, along with expression levels in matched normal samples and their non-silent tumor mutational burden (TMB). In general, 12-CK scores corresponded with TMB. ACC, adrenocortical carcinoma; BLCA , bladder carcinoma; BRCA , breast carcinoma; CESC, cervical squamous carcinoma; CHOL , cholangiocarcinoma; COAD, colon adenocarcinoma; ESCA , oesophageal carcinoma; GBM, glioblastoma; HNSC, head and neck squamous cell carcinoma; KICH, kidney chromophobe; KIRC, kidney renal clear- cell carcinoma; KIRP, kidney renal papillary cell carcinoma; LGG, lower- grade glioma; LIHC, liver hepatocellular carcinoma; LUAD, lung adenocarcinoma; LUSC, lung squamous cell carcinoma; MESO, mesothelioma; OV, ovarian serous cystadenocarcinoma; PAAD, pancreatic adenocarcinoma; PCPG, pheochromocytoma and paraganglioma; PRAD, prostate adenocarcinoma; READ, rectum adenocarcinoma; SARC, sarcoma; SKCM, skin cutaneous melanoma; STAD, stomach adenocarcinoma; TGCT, testicular germ cell tumor; THCA , thyroid carcinoma; UCEC, uterine corpus endometrial carcinoma; UCS, uterine carcinosarcoma; UVM, uveal melanoma. *p < 0.05, **p < 0.01, ***p < 0.001, ****p < 0.0001.

Based on our previous work in colorectal cancer, Messina et al. interrogated the 12-CK score on 14,492 distinct primary and metastatic solid tumors housed within the Moffitt Cancer Center biorepository (12). In this analysis, tumor samples harvested from the oral cavity, cervix, tongue, skin and lung were found to have the highest 12-CK scores. Expanding on our institutional cohort, we also compared the pattern of 12-CK expression across different tumor types using The Cancer Genome Atlas (TCGA) data (**Figure 1**), similar to the analysis done by Sautès-Fridman et al. (3, 4) but with normal samples added and mutational burden.

As noted by Sautès-Fridman et al. (3, 4), the 12-CK scores were highly heterogeneous across tumor types, both in level and range within a specific tumor type. The range is largest in bladder cancer (BLCA, interquartile range, IQR=11.3), cholangiocarcinoma (CHOL, IQR=10.4), sarcoma (SARC, IQR=9.1), and thyroid cancer (THCA, IQR=9.5). Heterogeneity of the 12-CK scores amongst these tumors suggest different degrees of immune activation and TLS formation. Secondly, 12-CK scores generally corresponded with the median tumor mutational burden (TMB) as previously described (46), (Spearman r=0.46, p=0.01) suggesting a link between the neoantigen burden and immune activation (47). A notable exception was the high 12-CK scores affiliated with minimally mutated testicular germ cell tumors (TGCT). Corroborating these findings, Klein et al. (48) found TLS to be prominently featured only within the microenvironment associated with testicular seminoma, but not that of tissues harvested from patients with other benign testicular pathologies. The etiology behind TLS induction in such a mutationally silent tumor remains to be seen. Finally, 12-CK scores were higher in tumor samples than normal controls, in esophageal cancer (ESCA), clear cell renal cell carcinoma (KIRC), breast cancer (BRCA), stomach cancer (STAD), and head and neck squamous cancer (HNSC). On the other hand, thyroid cancer (THCA), liver hepatocellular carcinoma (LIHC), and colorectal cancer (READ & COAD) demonstrated lower 12CK score than benign counterparts (**Figure 1**).

### 12-CK Score Identifies TLS and Is Linked to Prognosis

**Figure 2:**
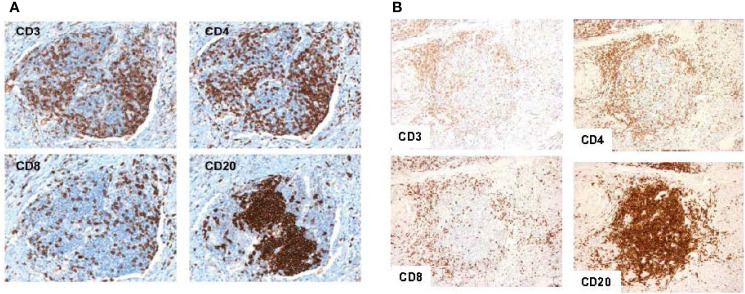
Representative tertiary lymphoid structures found in 12-CK high samples. Typical tertiary lymphoid structure with CD20+ B cells concentrated as a follicle, and CD3+, CD4+, and CD8+ T cells appearing in the parafollicular cortex or marginal zones and with some dispersion into the follicle in a melanoma tumor sample **(A)**. Similar patterns of cellular distribution and structure are recapitulated in breast tumor samples. (Adapted from Messina et al., *Sci Rep*, 2012; Prabhakaran *et al*., *Breast Cancer Res*, 2017). Similar patterns of cellular distribution and structure are recapitulated in breast tumor samples **(B)**.

Since the seminal work in colorectal cancer, our group has further elucidated the implications of the 12-CK score in various other tumor types. In a cohort of 120 samples collected from metastatic lesions in patients with stage IV melanoma, Messina et al. found that patients with low 12-CK scores contained minimal to absent peritumoral lymphocytic infiltrate (12). Conversely, patients with the highest 12-CK scores all had a marked peritumoral lymphocytic host response, punctuated by an abundance of TLS. These TLS contained prominent lymphoid follicles containing CD20^+^ B cells, with CD3^+^ T cells clustered within the parafollicular cortex like zones. While CD86^+^ activated T cells were diffusely present within these structures, FoxP3^+^ T regs were excluded (**Figure 2**). Additionally, in a limited cohort of 10 patients, the presence of TLS was associated with increased survival. Of particular interest, a single patient with prolonged partial response to ipilimumab (CTLA4 blockade) exhibited TLS within their tumor sample.

In another cohort of 366 patients with breast cancer, high 12-CK scores correlated with Caucasian race (p=0.03), poorly differentiated/high grade tumors (p<0.0001), and were more likely to be ER/PR negative and HER2 positive (p=0.001) (49). In addition, higher 12-CK tumors tended to be of the basal and HER2-positive molecular subtypes as classified by PAM50. More importantly, high 12-CK scores were associated with superior RFS (HR = 0.85, p = 0.018) and OS (HR = 0.63, p < 0.01). By molecular subtypes, both basal- and HER2-subtyped patients derived survival benefit from having a high 12-CK gene expression. H&E and immunohistochemistry staining of the 12-CK high tumors yielded similar results as those seen in colorectal carcinoma and melanoma (i.e. lymphocytic aggregates with CD20^+^ B cells concentrated as a follicle, and adjacent CD4^+^ and CD8^+^ T lymphocytes). Furthermore, tumors expressing high 12-CK scores also expressed genes related to immune activation, including BTLA, D274, CD69, CTLA-4, granzyme B, and IFN-γ.

### 12-CK in Bladder Cancer

**Figure 3:**
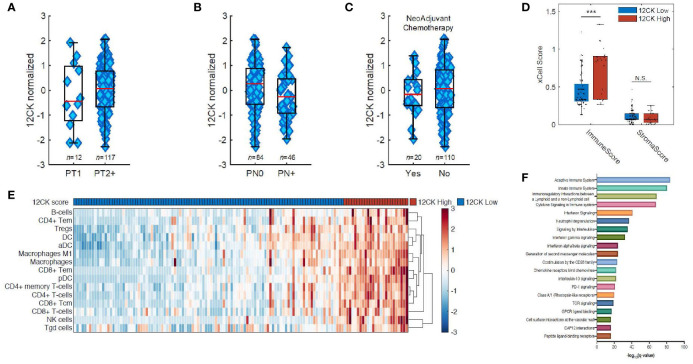
Implications of the 12-chemokine score. The 12-CK scores were found to be independent of traditional prognostic indicators, such as pathologic **(A)** T-staging and **(B)** N-staging, as well as to **(C)** the receipt of neoadjuvant chemotherapy. **(D)** High 12-CK scores were furthermore related to elevated immune scores, but not stromal scores as delineated by the gene signature-based deconvolution method xCell. **(E)** Furthermore, 12-CK high tumors highly expressed signatures related to both innate and adaptive immune cells. **(F)** On gene set enrichment analysis, high 12-CK tumors expressed gene sets related to TCR signaling, CD28 co-stimulation, IFN-γ signaling, IFN-α/β signaling, cytokine signaling, chemokine receptor binding and neutrophil degranulation. ***p < 0.001. NS, Non-significant.

Grounded in these works, we hypothesized that TLS played an integral role in promoting effective anti-tumor immunity in the context of bladder cancer. We collected 130 muscle invasive bladder cancer (MIBC) samples for Affymetrix microarray analysis. The 12-CK score was not found to correlate with traditional prognostic indicators such as pathologic T-staging or N-staging (**Figures 3A, B**). Moreover, no differences were observed in the 12-CK score amongst tumors that were treatment naïve *vs.* those collected following neoadjuvant chemotherapy (**Figure 3C**) (50).

To further explore the immunologic correlates of high 12CK expression, a cell type enrichment analysis from gene expression (xCell) was used to deconvolute the makeup of the TME in the mRNA microarray data. Cell type enrichment scores across 64 immune and stromal cell types were obtained. Although stromal scores were similar between the two cohorts, immune scores representing the overall immune cell content were markedly higher in the 12-CK High tumors (**Figure 3D**). The 12-CK High tumors expressed transcriptomic signatures associated with CD4^+^ T lymphocyte, CD8^+^ T lymphocyte, activated dendritic cells (aDC), and B lymphocytes. Furthermore, M1 macrophage, Macrophages, NK cells, CD8^+^ Tem, CD4^+^ Tem, and B cells were enriched in 12-CK High tumors, suggesting both a heightened innate and adaptive immune response (**Figure 3E**) (50).

These results were corroborated by findings from gene set enrichment analysis using the REACTOME gene sets, where gene sets associated with both the innate and adaptive immune response were found to be elevated in the 12CK-High tumors. In addition, other gene sets associated with immune activation including TCR signaling, CD28 co-stimulation, IFN-γ signaling, IFN-α/β signaling, cytokine signaling, chemokine receptor binding and neutrophil degranulation were correspondingly found to be elevated in the 12CK-High tumors (**Figure 3F**) (50).

**Figure 4:**
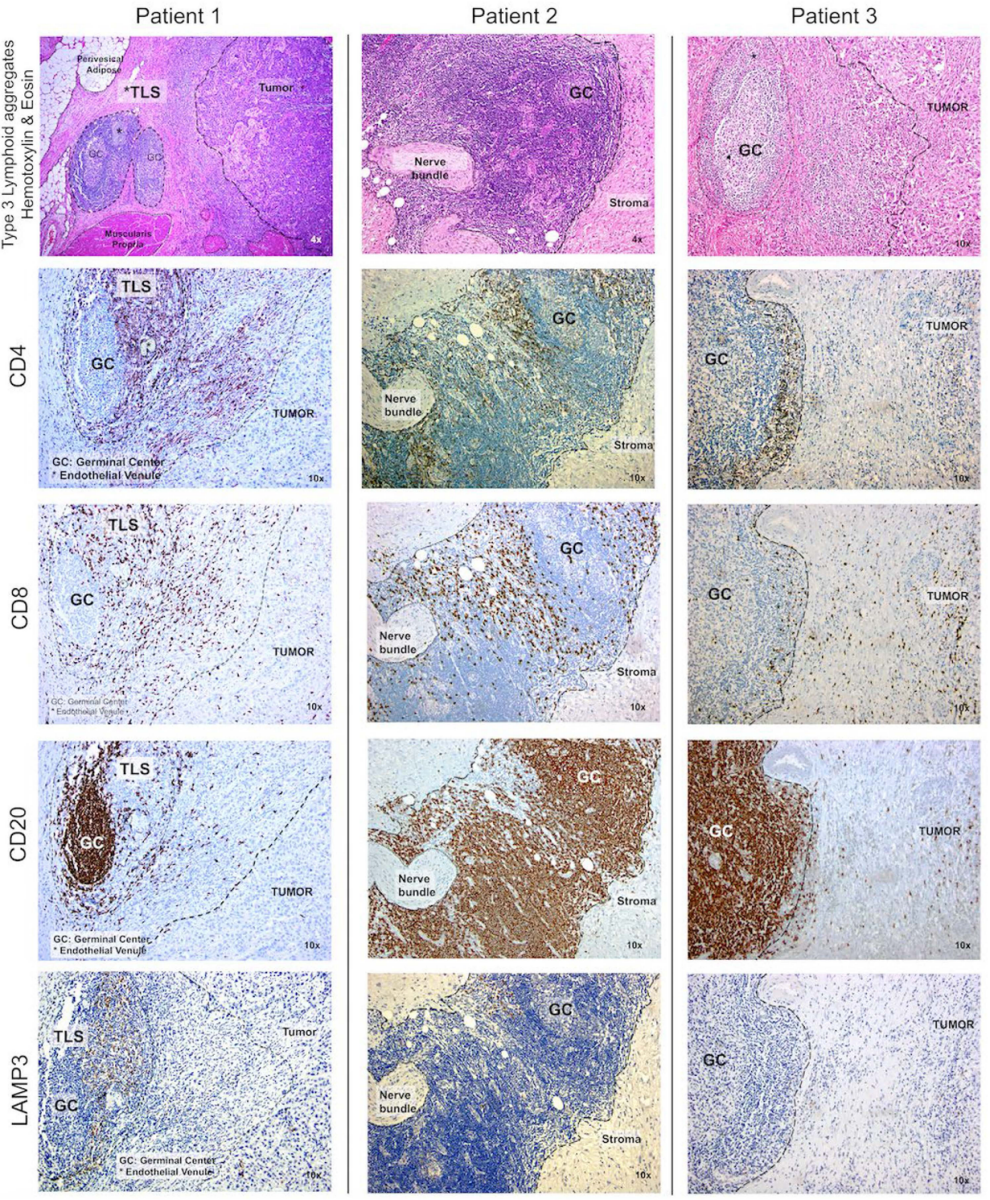
High 12-chemokine scores correlated with higher densities of tumor infiltrating immune cells. Immunohistochemistry was performed using antibodies to CD4, CD8, CD20, and LAMP3 to mark CD4+ T cells, CD8+ T cells, B cells, and activated dendritic cells, respectively. Compared to samples with low 12-CK scores, the 12-CK high tumors were found to contain Type 3 TLS with prominent germinal centers, along with increased infiltrating CD4+ T cells, CD8+ T cells, CD20+ B cells, and comparable levels of activated dendritic cells.

To confirm these findings, immunohistochemistry (IHC) was performed using antibodies to CD4, CD8, CD20, and LAMP3, and cellular densities were quantified using the H-score. Within their TME, 12-CK High tumors consistently exhibited a more robust immuno-environment marked by a higher density of CD4^+^ T cells (p=0.002), CD8^+^ T cells (p<0.001), and CD20^+^ B cells (p=0.002), but not LAMP3^+^ aDC (p=0.3) (**Figure 4**). Next, we systematically identified the presence of TLS in the TME of 12-CK High *vs.* 12-CK Low tumors and classified them into Type I-III as previously described (19). Of the 23 12-CK High tumor samples evaluated, there were 11 with Type III TLS. In contrast, Type III TLS was only found in 1 of the 21 12-CK Low tumor samples (p<0.002) (50).

Kaplan-Meier survival analyses of the TCC130 cohort revealed improved progression-free survival (PFS, HR 0.29, p=0.004), disease-specific survival (DSS, HR 0.29, p=0.004), and overall survival (OS, HR 0.55, p=0.03) amongst 12CK-High patients (51). On multi-variable analysis incorporating age, pathologic T and N stage, and use of neoadjuvant chemotherapy, high 12-CK score was found to independently prognosticate improved PFS (HR 0.77, 95% CI 0.62-0.95, p=0.01), DSS (HR 0.63, 95% CI 0.49-0.81, p=0.0003), and OS (HR 0.81, 95% CI 0.65-0.998, p=0.048). To externally validate the prognostic value of the 12CK score, we interrogated data from TCGA, and found similar improvements in PFS (HR 0.55, p=0.007), DSS (HR 0.40, p=0.002), and OS (HR 0.59, p=0.01) in 12CK-High patients. Together, these findings highlight the important favorable prognostic implication of high 12CK-High scores in surgically treated MIBC patients and corroborates findings by other groups on the important prognostic implications of tumor-associated CD38+ plasma cells and TLS in bladder cancer (52).

In summary, 12-CK scores vary widely between different tumor types and within specific tumor types. In general, high scores were seen amongst cancers known to have high TMB, presumably with high neoantigenic stimuli to trigger a strong immunogenic response. On histologic review, tumors marked by high 12-CK scores consistently demonstrated a robust peritumoral inflammatory response underpinned by the presence of TLS consisting of germinal centers rich in CD20^+^ B cells, plus an adjacent T-cell zone composed by CD4^+^ and CD8^+^ lymphocytes, and HEVs. Consistently across several tumor types, global high 12-CK scores were found to convey favorable oncologic outcomes.

## Predicting Response to Immunotherapy

Perhaps even more relevant than its ability to prognosticate, the 12-CK score may also serve as a predictive biomarker for response to various modalities of anticancer therapies. Multiple studies have linked pathological complete response (pCR) following neoadjuvant chemotherapy in breast cancer to the presence of TLS. In a cohort of 1,058 patients, high densities of B cells in the context of TLS was found to correlate with pCR following combination neoadjuvant chemotherapy (53). Similar findings were recapitulated in another study consisting of 108 triple-negative breast cancer patients following neoadjuvant chemotherapy, in whom higher densities of HEVs, CD20^+^ B cells and TLS were significantly associated with disease-free survival following surgery (54). In the adjuvant setting, high densities of TILs and TLS have also been demonstrated to confer favorable response to chemotherapy in bladder cancer (20) and to trastuzumab in hormone receptor-negative, HER2^+^ breast cancer (55).

Intriguingly, Messina et al. also uncovered a possible link between high 12-CK scores and response to immunotherapy. A Stage IV metastatic melanoma patient with tumor highly expressive of the 12-CK score and TLS enrichment demonstrated a more than 30 month partial response to ipilimumab (CTLA-4 antagonist) (12). Similar findings were repeated in three recent publications spanning ICB clinical trials in melanoma, renal cell carcinoma and soft tissue sarcoma (14–16), in which the presence of TLS in tumor samples were consistently associated with improved survival following therapy. The exact mechanism through which the humoral response generated from the B-cell rich TLS contributes to the overall antitumor immunity in the context of ICB treatment is unknown. Moreover, as ICB is thought to confer its antitumor effects primarily through the T cell compartment, its augmented efficacy in the B-cell enriched TME is counterintuitive.

Nevertheless, some mechanistic insights were uncovered within the three aforementioned studies (14–16). Using CyTOF, Helmink et al. (15) found memory B cells and plasma cells to be more abundant in the TME of ICB responders *vs.* non-responders. Using spatial high-plex proteomic analysis, Cabrita et al. (14) found more CD4^+^ than CD8^+^ T cells within or in close proximity to the TLS in metastatic melanoma samples. T cells found in the vicinity of TLS were highly expressive of the pro-survival anti-apoptotic marker BCL-2, signifying antigen-specific activation (56). Interestingly, the T cells found in tumors without TLS had increased expression of immune checkpoint receptors PD-1 and TIM3 as well as lower levels of the anti-apoptotic marker BCL-2, suggesting immune exhaustion (56). Using single cell RNA sequencing, B cell rich samples (presumably with enrichment of TLS) were confirmed to contain more CD4^+^ and CD8^+^ T cells with naïve and/or memory-like phenotypes. These findings were corroborated in the setting of soft tissue sarcoma in an independent study by Petitprez et al. (16) TLS containing tumors were found to have higher densities of infiltrating CD3^+^ T cells, CD8^+^ T cells and CD20^+^ B cells, even while controlling for the T and B lymphocytes found within the TLSs themselves. The TLSs were also found to contain CD4^+^PD1^+^CXCR5^+^ T follicular helper cells, CD23^+^CD21^+^ follicular dendritic cells, and PNAd^+^ HEVs.

Further details on the cross-talk between the T- and B-lymphocytes in the context of ICB were uncovered by experiments using a murine triple negative breast cancer model (57). The generation of T cell memory following ICB treatment was critically dependent on B cell activity. Vice versa, tumor infiltration by B cells also hinged on concurrent T_fh_ cell activation and Regulatory T cell (Treg) inhibition. B cell activation following ICB treatment led to proliferation of classed switched plasma cells and increased production of tumor specific IgGs. Moreover, blockade of the T_fh_ cytokine IL21 completely abrogated B cell activation and therapeutic response from anti-CTLA4 therapy (57). In addition, our group has recently established the critical role of T_fh_ in initiating the formation TLS (Chaurio et al., unpublished). In sum, these studies outline a complex web of interactions between T- and B-lymphocytes following ICB treatment, with a particular focus on the importance of TLS in conferring therapeutic efficacy.

Supported by these mechanistic insights, transcriptomic signatures have been proposed by various groups as surrogate markers for the presence of TLS/B cells to predict response to ICB. Cabrita et al. constructed a signature using hallmark TLS-related genes (*CCL19, CCL21,CXCL13, CCR7, CXCR5, SELL* and *LAMP3*) (14), while Helmink et al. resorted to B cell related genes (*MZB1, JCHAIN, IGLL5, FCRL5, IDO1, IFNG* and *BTLA*) (15). While both groups were able to differentiate responders from non-responders within their respective ICB trials, these signatures have not yet been tested in ICB trials involving other tumor types.

Given that the 12-CK score has been successfully deployed to both reflect the presence of TLS, and prognosticate for improved survival in multiple tumor types (12, 25, 49), we also examined its predictive role for response to ICB. We used data from a recently completed ICB trial in metastatic bladder cancer. Publicly available RNA-seq data from the IMvigor 210 study (58) was extracted. In this single-arm, phase 2 trial, patients with inoperable locally advanced or metastatic bladder cancer with disease progression following platinum-based chemotherapy were enrolled and treated with intravenous atezolizumab (1200mg, given every 3 weeks). In 310 patients receiving atezolizumab treatment, 15% objective response was observed overall, with ongoing responses observed in 84% of the responders. Stratified by treatment response, the complete responders (CR) exhibited significantly higher 12-CK scores than all other groups. Strikingly, the 12-CK High signature conferred a median overall survival benefit of almost 1 year in the atezolizumab-treated patients (51). Similar results using the 12-CK score have also been reported in clinical trials using PD-1 inhibitor in similar patients (59).

## Conclusion and Future Directions

Immunotherapy has emerged as a fourth pillar in the treatment of cancer along with surgery, radiotherapy and chemotherapy. By directing the body’s immune system to target cancer cells, immunotherapy has the advantages of reducing toxicity while conferring long term response. Despite the success with treatments such as ICB, its underlying mechanisms of action remain incompletely understood. To date, research in immuno-oncology has been sharply focused on the T-cell compartment. However, as efficacy rates to immunotherapy continue to stubbornly stagnate, attention has been divested to uncover the function of other immunologic cell types known to be key players in various immunogenic processes.

The discovery of TLS and their strong link to improved prognosis in multiple cancer types has shed light on the function of B cells in the context of TME. It is increasingly understood that these structures are emblems of not only a robust, but effective local anti-tumor response. As such, they may serve as reliable biomarkers for improved prognosis and/or response to immunotherapy. However, as the density, distribution and location of TLS in the TME are extremely variable, an easily attainable surrogate biomarker is needed to quantify its presence and phenotypic characteristics. Ideally, this marker should be measured objectively and reproducibly with both internal and external validity. It should also be safe to implement (60).

As demonstrated in our previous studies of the 12-CK score in the prognosis of colorectal carcinoma (25), melanoma (12), breast carcinoma (49), and urothelial carcinoma (50, 51), high scores reliably recapitulated the presence of TLS across several tumor types and prognosticated for survival following standard-of-care therapies. However, whether the score can be refined to nuances of the TLS maturation stage and/or localization within the TME remains to be seen. In addition, we are also discovering the predictive value of the 12-CK scores in the context of immunotherapy, such as ICB. Given the wide adoption of next-generation sequencing in the management of various cancers, the 12-CK score is easily attainable from tissue samples obtained for diagnostic purposes prior to the start of treatment. It may also serve to complement the existing biomarkers, such as TMB and PD-L1 status, to form a predictive nomogram used to refine selection of patients for treatment success.

On the other hand, measurement of the induction of TLS by various immunotherapeutic agents may be used to track treatment efficacy. In a clinical trial combining ipilimumab and nivolumab for preoperative treatment in locoregionally advanced urothelial cancer, responders were not necessarily found to have higher TLS in their pre-treatment samples, but rather had a higher degree of TLS induction while on-treatment (18). As repeated tissue sampling may be impractical during the treatment course, “liquid biomarkers” easily collected from the serum may be used as an alternative method to monitor response. To that end, detection of increasing levels of serum CXCL13 has been demonstrated to signify germinal center activity and broadly elevated antibody production (10, 61). Whether CXCL13 measured from the serum alone can serve to track the formation of TLS in the context of immunotherapy in cancer awaits investigation.

Finally, as more details of the B-cell mediated anti-tumor response continue to be unraveled, novel therapeutic strategies to complement ICB will undoubtedly emerge. As the key orchestrators of an effective anti-tumor immune response, TLS are front and center as the potential targets for therapeutic modification. In turn, the 12-CK score may serve as a potential biomarker to predict for response or track efficacy in many of these novel strategies, and serve as an indispensable tool for immuno-oncologists as we launch into the next phase of innovation.

## Author Contributions

RL: Study concept and manuscript composition. AB: Manuscript composition and critical revision. LZ: Critical revision. JD: Critical revision. RP: Critical revision. YK: Critical revision. RJ: Critical revision. GG: Critical revision. JC-G: Critical revision. JM: Study concept, critical revision, and supervision. All authors contributed to the article and approved the submitted version.

## Funding

RL: Funding provided by the Clinical Science Division at Moffitt Cancer Center and the Campbell Family Foundation. AB and RP: This work has been supported in part by the Biostatistics and Bioinformatics Shared Resource at H. Lee Moffitt Cancer Center and Research Institute, a NCI designated Comprehensive Cancer Center (P30-CA076292). JJM: Funding provided by NCI-NIH (1R01 CA148995, 1R01 CA184845, P30 CA076292, and P50 CA168536), Cindy and Jon Gruden Fund, Chris Sullivan Fund, V Foundation, and the Dr. Miriam and Sheldon G. Adelson Medical Research.

## Conflict of Interest

RL: Clinical trial protocol committee - Cold Genesys, BMS; Scientific advisor/consultant – BMS, Fergene. JM: Associate Center Director at Moffitt Cancer Center, has ownership interest in Fulgent Genetics, Inc., Aleta Biotherapeutics, Inc., CG Oncology, Inc., Myst Pharma, Inc., Verseau Therapeutics, Inc., AffyImmune, Inc., and Tailored Therapeutics, Inc., and is a paid consultant/paid advisory board member for ONCoPEP, Inc., CG Oncology, Inc., Morphogenesis, Inc., Mersana Therapeutics, Inc., GammaDelta Therapeutics, Ltd., Myst Pharma, Inc., Tailored Therapeutics, Inc., Verseau Therapeutics, Inc., Iovance Biotherapeutics, Vault Pharma, Inc., Noble Life Sciences, and Fulgent Genetics, Inc., UbiVac, LLC, Vycellix, Inc., AffyImmune, Inc., and Aleta Biotherapeutics, Inc. A patent on the 12 chemokine gene expression signature in bladder cancer was issued on March 10, 2020, titled, *“Immune Gene Signatures in Urothelial Carcinoma (UC)”* (10,583,183). Inventors are: JM, Anthony M. Magliocco, and AB. A provisional patent application was filed on August 27, 2020, titled *“Immune Gene Signature in Muscle Invasive Bladder Cancer”* (Serial No. 63/071,320). Inventors are: RL, JM, and AB.

The remaining authors declare that the research was conducted in the absence of any commercial or financial relationships that could be construed as a potential conflict of interest.
